# Freeze–thaw *Caenorhabditis elegans* freeze–thaw stress response is regulated by the insulin/IGF-1 receptor *daf-2*

**DOI:** 10.1186/s12863-015-0298-5

**Published:** 2015-12-03

**Authors:** Jian-Ping Hu, Xiao-Ying Xu, Li-Ying Huang, Li-shun Wang, Ning-Yuan Fang

**Affiliations:** The Department of Geriatrics, Ren-Ji Hospital, Shanghai Jiao-Tong University School of Medicine, Shanghai, China; The Division of Translational Medicine, Minhang Hospital, Fudan University, Shanghai, China

**Keywords:** *C. elegans*, Freeze–thaw stress response, Insulin/IGF-1 receptor *daf-2*, Transcription factor *daf-16*/FOXO

## Abstract

**Background:**

Adaption to cold temperatures, especially those below freezing, is essential for animal survival in cold environments. Freezing is also used for many medical, scientific, and industrial purposes. Natural freezing survival in animals has been extensively studied. However, the underlying mechanisms remain unclear. Previous studies demonstrated that animals survive in extremely cold weather by avoiding freezing or controlling the rate of ice-crystal formation in their bodies, which indicates that freezing survival is a passive thermodynamic process.

**Results:**

Here, we showed that genetic programming actively promotes freezing survival in *Caenorhabditis elegans*. We found that *daf-2*, an insulin/IGF-1 receptor homologue, and loss-of-function enhanced survival during freeze–thaw stress, which required the transcription factor *daf-16*/FOXO and age-independent target genes. In particular, the freeze–thaw resistance of *daf-2*(rf) is highly allele-specific and has no correlation with lifespan, dauer formation, or hypoxia stress resistance.

**Conclusions:**

Our results reveal a new function for *daf-2* signaling, and, most importantly, demonstrate that genetic programming contributes to freezing survival.

**Electronic supplementary material:**

The online version of this article (doi:10.1186/s12863-015-0298-5) contains supplementary material, which is available to authorized users.

## Background

Cold temperature is a critical environment stimulus to animals. Subzero temperatures especially may adversely affect animals by direct lethal effects and damage caused by ice formation [1]. The ability of animals to sense and respond to cold temperatures, even below freezing, is essential for survival is cold environments. Freezing is also widely used for many medical, scientific, and industrial purposes, such as strain preservation and organ preservation. Understanding how to enhance survival and maintain normal physiological functions in the presence of freeze stress is critical for animals in nature and human research.

Natural freezing survival in animals has been extensively studied. Previous studies demonstrated that animals survive in extremely cold weather by avoiding freezing or controlling the rate of ice-crystal formation in their bodies [2], which indicates that freezing survival is a passive thermodynamic process. However, response to freeze–thaw stress and other biological phenomena, such as longevity and hypoxia resistance, may be genetically programmed. Because it is a powerful model in molecular genetics, the nematode *Caenorhabditis elegans* is suitable for studying genetic response to freeze–thaw stress.

Insulin/insulin-like growth factor 1 (IGF-1)-like signaling is the best-characterized pathway that regulates the lifespan and other stress-resistance traits of *C. elegans*. The insulin/IGF-1 receptor homologue *daf-2* activates a conserved phosphatidylinositol-3-OH kinase (PI(3)K)/3-phosphoinositide-dependent kinase-1 (PDK1)/Akt signal transduction pathway, which prevents FOXO transcription factor *daf-16* entry into the nucleus [3–5]*. daf-2* reduction/loss-of-function (rf) produces a longer lifespan [6]. Conversely, the PTEN phosphatase homologue, *daf-18*(rf), suppresses life-span extension induced by *daf-2*(rf) [7, 8].

Insulin/IGF-1 signaling is also involved in formation of dauer larvae, which have an alternative, developmentally arrested third larval stage (L3) [9]. In unfavorable environments, such as crowding or food shortage, insulin/IGF-1 signaling or transforming growth factor-beta (TGF-β) is suppressed; unliganded nuclear receptor DAF-12 regulates dauer diapause [10]. Insulin/IGF-1 signaling is also involved in other stress tolerances, including oxidative stress, ultraviolet light, heat shock, or hypoxia stress. The longevity and stress tolerance produced by insulin/IGF-1 signaling mutants require nuclear translocation or nuclear activity of *daf-16* [11]. Savory et al. [12] showed that *daf-16* is important for delta-9 desaturase gene expression, which is important for survival at low temperatures. Ohta [13] showed that the insulin-signaling pathway in the intestines and neurons is essential for temperature experience-dependent cold tolerance in animals. Animal survival in freezing conditions is a more complicated phenomenon than in cold temperatures without ice formation. Organs can be injured during freezing by physical factors, such as ice-crystal formation, dehydration, and cold [14]. Moreover, animals can also suffer biochemical damage, such as oxidative stress or hypoxia stress. [15]

To date, few studies have addressed the genes that regulate freezing tolerance or survival. We investigated the roles of *daf-2*(rf) in freeze–thaw stress-response regulation. We exposed *daf-2(e1370)* and other rf strains to freeze–thaw stress to identify *daf-2*(rf)-improved freeze-induced mortality and cell damage. Then, we tested other *daf-2*(rf) alleles with various phenotypic severities and performed molecular analysis to determine which signaling is involved. We performed the experiments through both genetic and morphological analyses with different *C. elegans* mutants.

## Results

### Insulin/IGF-1 receptor homologue *daf-2*(rf) regulates freeze–thaw stress survival

**Fig. 1:**
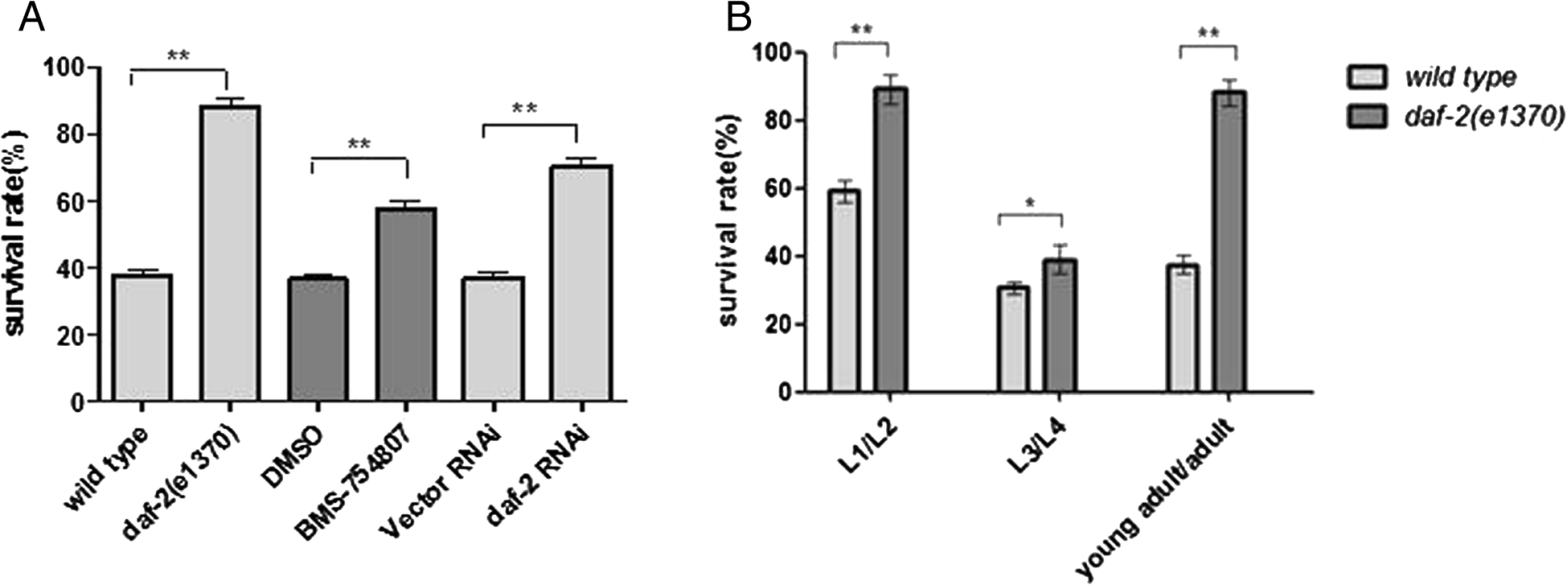
Enhanced freeze–thaw stress survival induced by IGF-1 receptor/*daf-2* reduction-of-function. **a** *daf-2(e1370*rf*)*, IGF-1 receptor inhibitor (BMS-754807) treatment, and *daf-2* RNAi increased freeze–thaw stress survival compared with corresponding wild-type control animals. Every assay was repeated five times (n > 170–203) in the same condition. Statistical significance was assessed by the Mann–Whitney test. **p < 0.01. **b** *daf-2(e1370)* enhanced freeze–thaw stress survival at different growth stages, from L1 to adult stages. Survival rates were assayed 6 h after recovering from freeze–thaw stress (L1/L2- and L3/L4-stage animals were exposed to freezing for 16 min, and young adult/adult animals were exposed to freezing for 8 min at −80 °C). Every assay was repeated five times (n > 180–286). Statistical significance was assessed by the Mann–Whitney test. *p < 0.05, **p < 0.01

To investigate the role of *daf-2*(rf) in freezing tolerance, we evaluated the survival rate of *daf-2(e1370)* and wild-type (*N2*) strains exposed to freeze–thaw stress. Two-day-old adults were exposed to -80 °C for 8 min and then thawed in a water bath at 30 °C; results showed that the *daf-2(e1370)* strain had a significantly increased survival rate compared with the *N2* strain (p < 0.01; Fig. 1a). To confirm the results of enhanced freezing survival produced by the reduction of *daf-2*/insulin-like signaling, we evaluated the survival rate of *N2* animals with *daf-2* RNAi interference or IGF_1_R inhibitor treatment. Animals with both *daf-2* RNAi inactivation and IGF_1_R inhibitor treatment had enhanced survival rates after freeze–thaw treatment (p < 0.01; Fig. 1a).

At high freezing rates, intracellular freezing occurs, which can lead to cell damage, mainly by ice-crystal formation [15, 16]. To reduce ice-crystal formation and cell damage, cells and organisms can be cryopreserved by slowly lowering the temperature until deep freezing temperature. *daf-2(e1370)* and *N2* animals were gradually cooled at a rate of about −1 °C/min (Additional file 1: Figure S1). Moreover, we also found that *daf-2(e1370)* animals had higher survival than *N2* at any growth stage. This result indicates that survival of *daf-2*(rf) animals following freeze–thaw damage is not stage- or age -specific (Fig. 1, Additional file 1: Figure S1A–C). We also found that the survival rate of L3/L4-stage worms was dramatically decreased compared with L1/L2-stage worms (Fig. 1b, Additional file 1: Figure S1A–B). This result indicates that freezing tolerance changed with development and age.

### Freezing-induced behavioral defects and cell defects blocked by *daf-2*(rf)

**Fig. 2:**
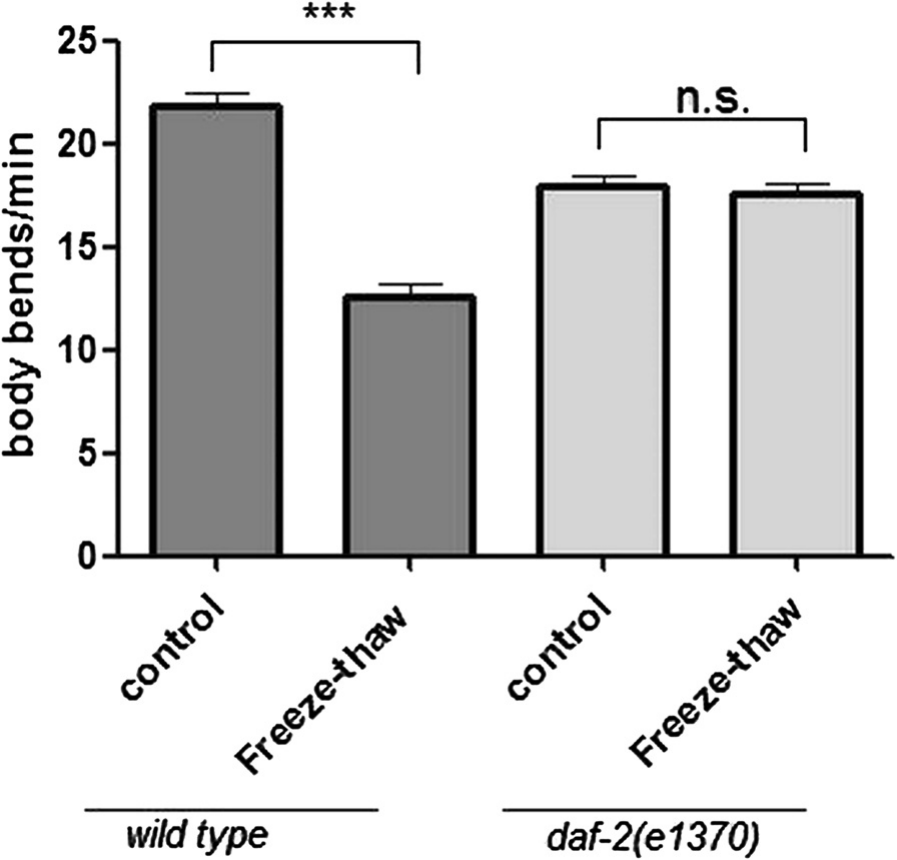
Behavioral effects of freeze–thaw stress in wild-type (*N2*) and *daf-2(e1370*rf*)* strains. Locomotion rate was quantified as the number of body bends per min after recovery from freeze–thaw stress. Locomotion of N2 (n = 10) was significantly reduced after recovery from freeze–thaw stress; *daf-2(e1370)* remained unchanged (n = 10). Statistical significance was assessed by the Mann–Whitney test. ***p < 0.001, n.s. - not significant

Maintaining physiological functions in freezing temperatures is challenging; therefore, evaluation of response to different freezing conditions is important. Under freeze–thaw conditions, animals frozen at 0 °C and thawed in 30 °C water baths did not differ in survival rate (Additional file 2: Figure S2.), which indicates that the animals’ physiological function loss is mainly caused by freezing rather than thawing. *daf-2(e1370)* animals survived and fully recovered locomotion after freeze–thaw stress. *N2* animals displayed significant locomotion defects after recovery from some stress (Fig. 2).

**Fig. 3:**
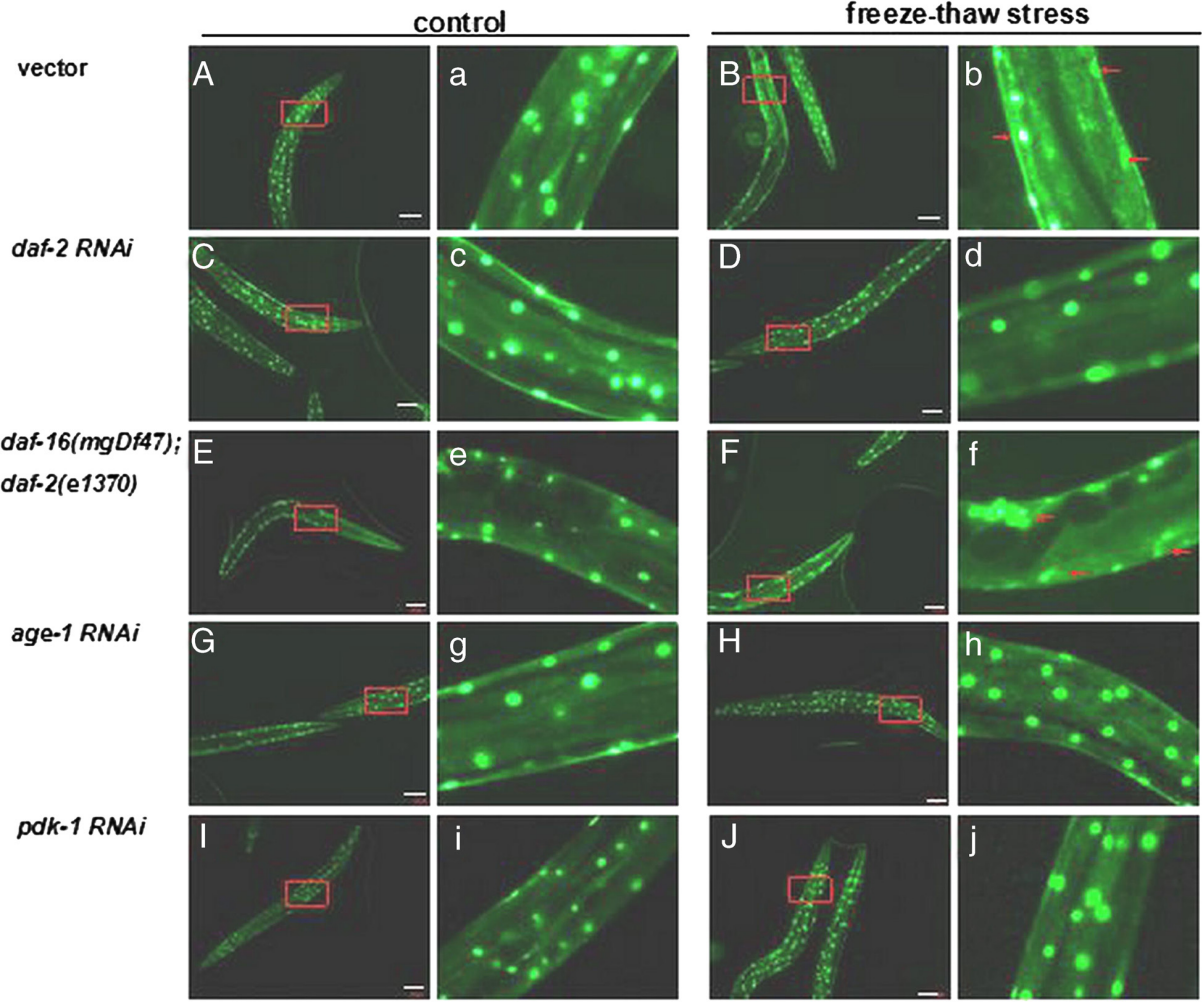
Freeze–thaw stress-induced morphologic cell defects blocked by *daf-2*(rf). Adult animals were treated with freeze–thaw stress as described in the Methods; after 6 h of recovery, surviving animals were scored for cell morphology. All GFP reporter genes [40] were stably integrated. Every assay was repeated three times (n > 12–21) in the same conditions. Nuclear-localized *pmyo-3:gfp* reporter gene expression in body wall muscle nuclei. *PD4251* (**A**, **a**), *daf-2* RNAi (**C**, **c**), [*daf-16(mgDf47)*; *daf-2(e1370)*] (**E**, **e**), *age-1* RNAi (**G**, **g**), or *pdk-1* RNAi (**I**, **i**) animals not exposed to freeze–thaw stress treatment were observed. *PD4251* (**B**, **b**) and [*daf-16(mgDf47)*; *daf-2(e1370)*] (**F**, **f**) animals’ nuclear GFP expression was fragmented (arrow) with freeze–thaw stress. Additionally, *daf-2* RNAi (**D**, **d**), *age-1* RNAi (**H**, **h**), and *pdk-1* RNAi (**J**, **j**) animals had preserved nuclear morphology with freeze–thaw stress

To investigate cell defects from freezing and protection by *daf-2*(rf), we examined the muscle cell morphology of animals exposed to freeze–thaw stress. Freezing caused striking nuclear fragmentation in muscle myocytes. RNAi inactivation of *daf-2* and downstream genes *age*, *pdk-1* maintained intact nuclei and protected myocytes from both nuclear fragmentation and death [Fig. 3].

### *daf-2* allelic specification for freeze–thaw stress survival is not a consequence of lifespan, dauer formation, or other stress resistance mechanisms

**Fig. 4:**
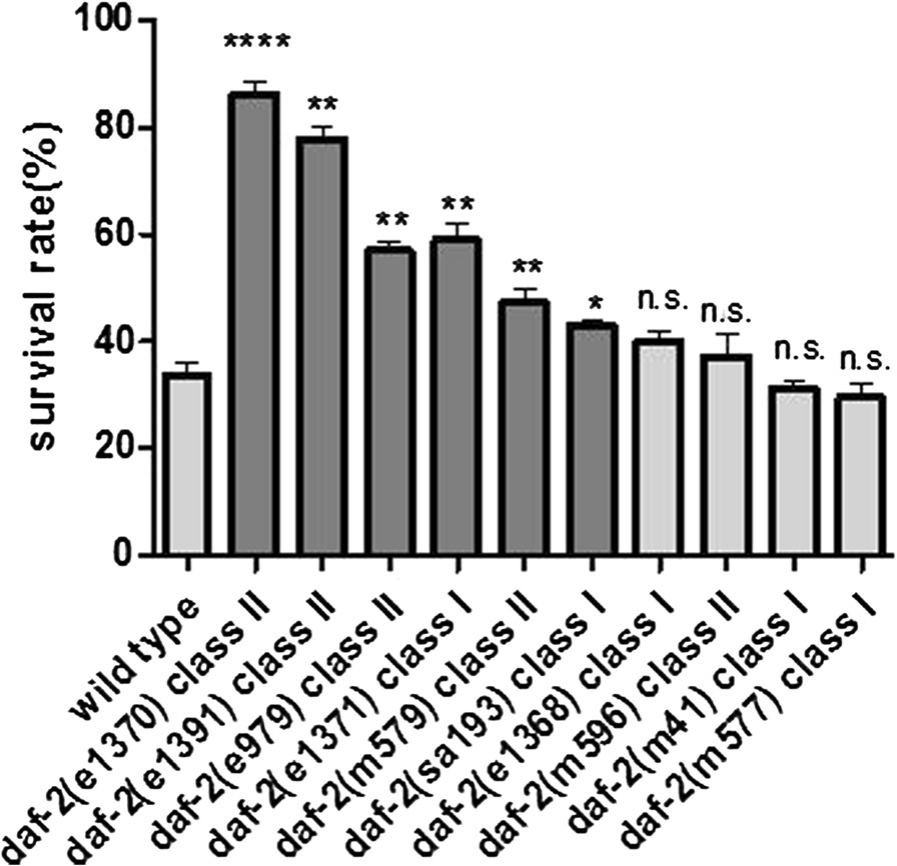
*daf-2*(rf) allelic variation for freeze–thaw stress survival. After freeze–thaw treatment, *daf-2(e1370)* had the highest survival rate followed by *e1391*, *e1371*, *e979*, *m579*, and *sa193*. Four strains (*e1368*, *m596*, *m41*, and *m577*) had no increased survival rate compared with wild-type (*N2*) animals. Survival rate analysis was repeated at least five times under similar conditions; mean lifespan values and statistical analyses of survival assays are shown in Additional file 5: Table S1. Statistical significance of differences between *daf-2*(rf) and *N2* strains was assessed by the Mann–Whitney test. *p < 0.05, **p < 0.01, ****p < 0.0001, n.s. - not significant

*daf-2*(rf) alleles tend to promote a prolonged lifespan and form dauer larvae. Consequently, *daf-2*(rf) mutants are always resistant to harsh environments [17]. To analyze whether freeze**–**thaw survival is correlated with lifespan, we tested freezing survival rates associated with 11 *daf-2* alleles’ (Fig. 4)*.* The freeze–thaw stress survival phenotypes were not well correlated with lifespan (r = 0.538; p = 0.088).

We found that *daf-2(e1370)* worms, which did not have the longest lifespan, had the highest freeze–thaw stress survival, followed by *e1391*, *e1371*, *e979*, and *m579*. Five alleles that were weak or produced no increased freezing survival had significantly increased lifespans as long as or longer than *e1370* [17]. Similarly, one allele that did not increase survival (*m41*) and two weaker alleles (*e1391* and *e979*) produced stronger Daf-c phenotypes than *e1370*, and one allele that did not increase survival (*e1368*) produced the same Daf-c phenotype as *e1370* [18, 19]. With regard to stress resistance, four *daf-2* alleles (*e1391*, *e979*, *e579*, and *m596*) were significantly resistant to thermal stress [18] but had weaker or no increased freeze–thaw survival. *daf-2* allele *e579* was significantly resistant to hypoxia stress but had weaker freeze–thaw survival. Three *daf-2* alleles (*e1371*, *e1391*, and *e979*) were weak or non-resistant to hypoxia stress but had higher freeze–thaw survival. *daf-2*(rf) freeze–thaw stress survival was highly allele-specific and did not appear to be a consequence of mechanisms that regulate lifespan, dauer formation, or other stress-resistance traits (Additional file 3: Figure S3).

### The insulin-signaling pathway, but not dauer signaling, longevity genes, or *trpa-1*, is essential for freeze–thaw stress survival

**Fig. 5:**
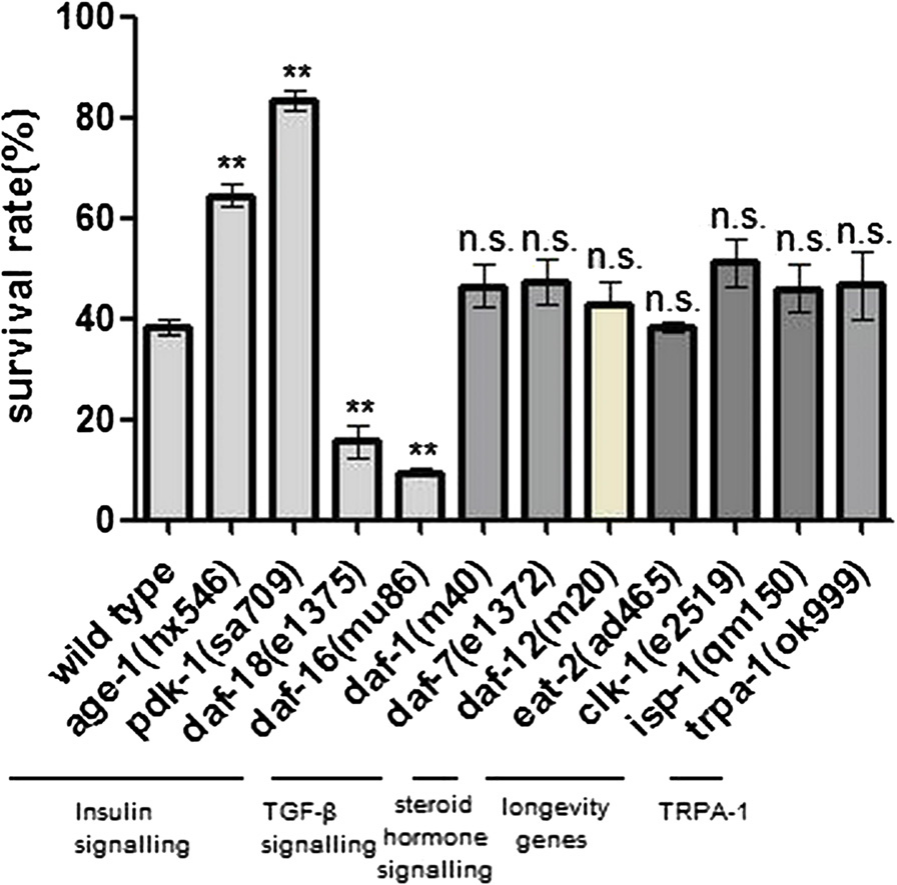
Insulin signaling regulates freeze–thaw stress survival. Freeze–thaw stress survival of mutants defective in insulin signaling, dauer formation signaling (TGF-β, steroid hormone signaling), longevity genes, and TRPA-1. Mutants defective in insulin signaling showed abnormal survival after freeze–thaw treatment. In contrast, TGF-β, steroid hormone signaling, other longevity gene, and *trpa-1* mutants showed normal survival rates compared with wild-type animals. Every assay was repeated at least three times (n > 110–297) in the same conditions. Statistical significance was assessed by the Mann–Whitney test. **p < 0.01, n.s. - not significant

**Fig. 6:**
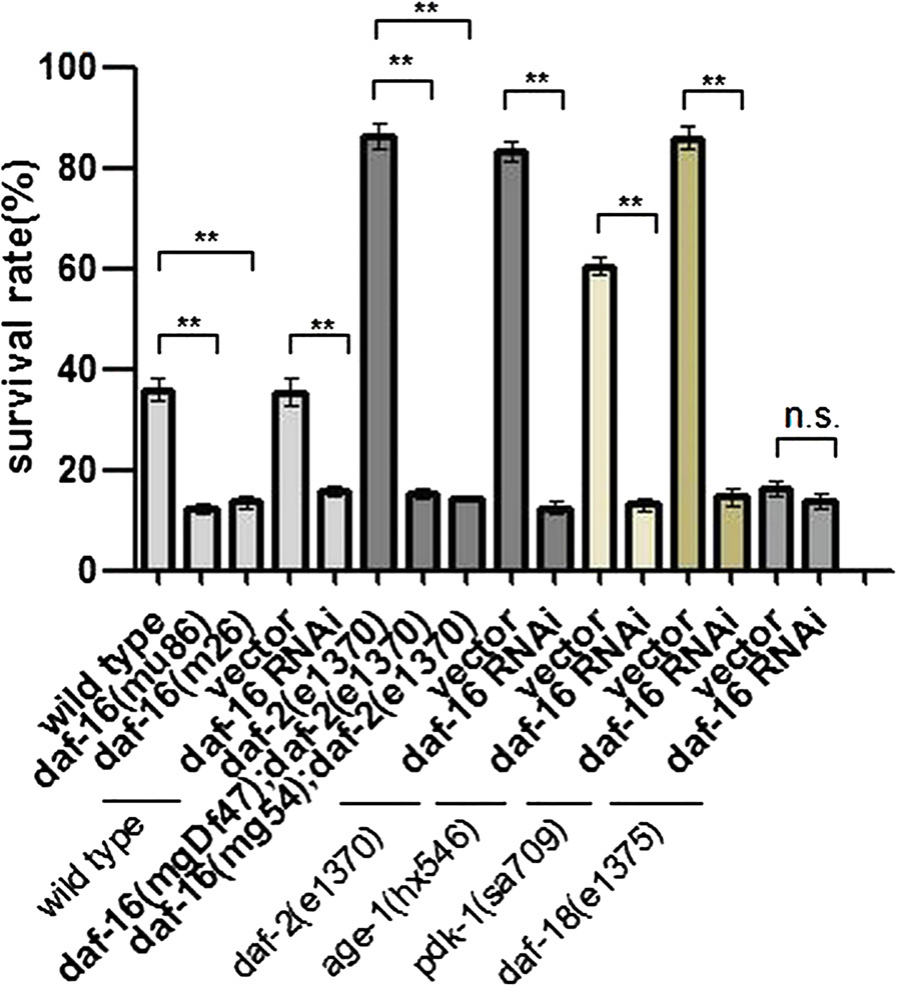
*daf-16* is essential for freeze–thaw stress survival. *daf-16* RNAi and *daf-16*-defective mutant loss enhanced survival of insulin signaling-defective mutants, including *daf-2(e1370)*, *age-1(hx546)*, and *pdk-1(sa709)*, and wild-type (*N2*). *daf-18*-defective mutants also had decreased stress survival after freeze–thaw treatment, similar to *daf-16* RNAi mutants. Every assay was repeated at least three times (n > 80–227) in the same conditions. Statistical significance was assessed by the Mann–Whitney test. **p < 0.01, n.s. - not significant

To determine the molecules downstream of the insulin receptor involved freeze–thaw stress survival, we tested various mutants defective in the known insulin-signaling pathway (Fig. 5). Phenotypic analysis showed that mutants defective in the *daf-2*/insulin receptor or its downstream molecules had abnormal enhancement or reduction of freeze–thaw stress survival (Fig. 5). Abnormal increments of freeze–thaw stress survival in *daf-*2 and downstream molecules mutants were suppressed by mutation or RNAi in the *daf-16*/FOXO-type transcriptional factor (Fig. 6).

Because the insulin-signaling pathway and other signaling molecules are essential for dauer larva formation, we tested other molecular components, including TGF-β and steroid hormonal signaling (Fig. 5) [19, 20]. However, we did not observe a considerable increase in these mutants (Fig. 5). The results indicate that the *daf-2*/insulin-signaling pathway, but not other dauer larva formation signaling, is essential for freezing survival.

*daf-2* is the best-characterized longevity gene in *C. elegans*. We tested other longevity genes mutants, including *eat-2*, *clk-1*, and *isp-1* mutants, and found that these animals did not have enhanced freezing survival compared with the *N2* strain. Recently, it was reported that the cold receptor transient receptor potential (TRP) channel, which is encoded by *trpa-1*, plays a central role in ageing and stress response to cold temperatures [21]. However, no reduced freeze–thaw survival was observed in *trpa-1* loss-of-function mutants. These results indicate that longevity genes and *trpa-1* are not essential for freeze–thaw stress survival.

### *daf-16* nuclear tranlsocation is not responsible for *daf-2*(rf)-enhanced freeze–thaw survival

**Fig. 7:**
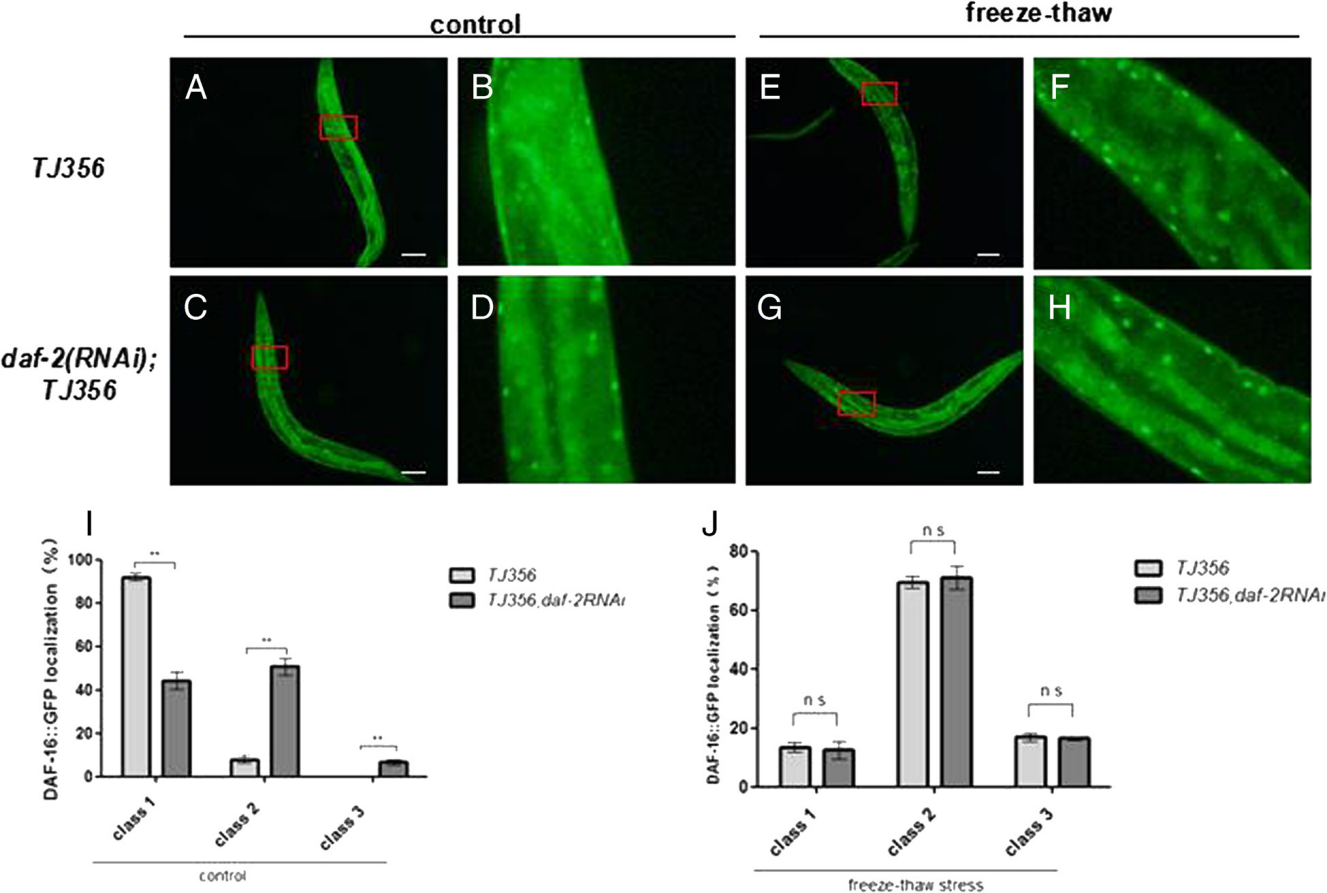
*daf-16* nuclear translocation does not enhance *daf-2*(rf) freezing survival. (**a**–**d**, **i**) *daf-2* RNAi enhanced *daf-16*::GFP nuclear expression compared with *TJ356* [41] animals (proportion *daf-16*::GFP nuclear translocation: class 1 = 0 %, class 2 > 0 %–50 %, class 3 > 50 %–100 %). (**e**–**h**, **j**) No difference in *daf-16*::GFP nuclear expression was found between *TJ356* and *daf-2(RNAi)* animals with freeze–thaw stress. Every assay was repeated at least three times in the same conditions. Statistical significance was assessed by the Mann–Whitney test. **p < 0.01, n.s. - not significant

To elucidate how *daf-16* affects *daf-2*(rf) enhanced freeze–thaw stress survival, cellular distributions of a *daf-16*::GFP fusion protein were first categorized on a scale of 1 (unlocalized) to 3 (fully nuclear localized) (Fig. 7). Distributions were scored in *daf-2* RNAi animals and compared with controls maintained at 20 °C. As a positive control, *daf-2* inactivation resulted in a marked translocation of *daf-16* to the nucleus (Fig. 7a–d, left histogram). With freeze–thaw stress, *daf-16* nuclear translocation significantly increased in both *daf-2* RNAi and control animals (Fig. 7e–h). However, the difference in *daf-16* nuclear translocation between *daf-2*(rf) and control animals disappeared, because there was no significant discrepancy in the subcellular localization of *daf-16*::GFP (Fig. 7, right histogram). These observations indicate that altering the subcellular localization of *daf-16* does not explain why *daf-2*(rf) has higher freeze–thaw stress survival than wild-type animals. We found that other mechanisms responsible for *daf-2*(rf) increased freezing survival.

To gather additional evidence to elucidate the nuclear activity of *daf-16* responsible for *daf-2*(rf) increased freeze–thaw stress survival, we assayed *daf-16* target genes. If the insulin pathway promotes *daf-16* nuclear activity, this pathway should regulate the expression level of *daf-16* target genes. We first examined the expression level of 21 direct transcriptional target genes of *daf-16* that are also involved in ageing, larval arrest, or fat formation. We found that mRNA levels of *C36A4.9* (*acs-19*, acetyl-CoA synthetase) and *C46A10.7* (*srh-99*, class H chemoreceptor/olfactory receptor) were markedly upregulated in *daf-2(e1370)* worms (Additional file 4: Figure 4). Mice deficient in the homologous AceCS2 (acetyl-CoA synthetase 2) gene cannot maintain normal body temperatures when starved or fed a LC/HF diet [22]. In *Drosophila*, central and peripheral elements of the olfactory receptor system are responsible for temperature adaptation [23].

**Fig. 8:**
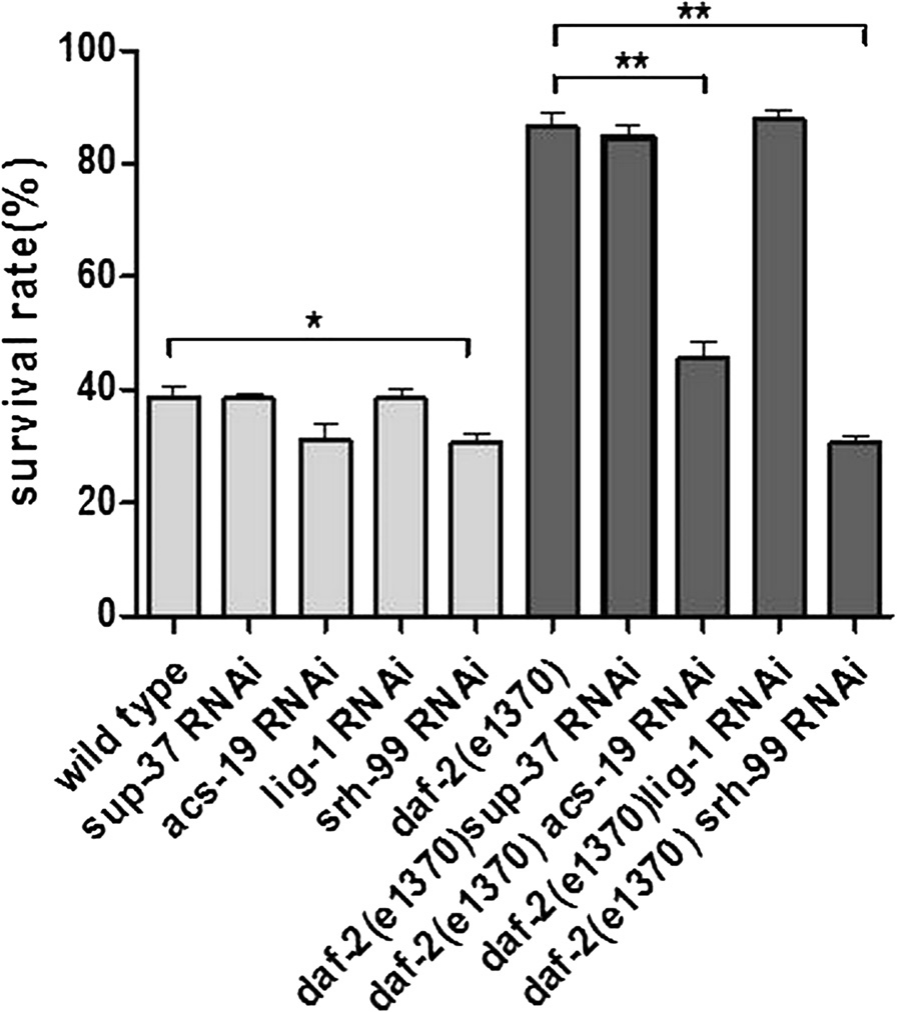
*daf-16* target genes *acs-19* and *srh-99* are required for *daf-2*(rf) enhanced freezing survival. *acs-19* and *srh-99* RNAi reduced *daf-2(e1370)* and wild-type animals’ freeze–thaw survival. *Sup-37* or *lig-1* RANi had no significant effects on daf-2(e1370) and wild-type worms’ freeze-thaw survival . Every assay was repeated at least five times (n > 150–187) in the same conditions. Statistical significance was assessed by the Mann–Whitney test. *p < 0.05, **p < 0.01

We infer that *acs-19* or *srh-99* may be required for freeze–thaw adaptation in *C. elegans*. For further elucidation, we assayed freeze–thaw survival rates with *acs-19* and *srh-99* RNAi in *daf-2(e1370)* and wild-type animals. We found that *acs-19* and *srh-99* RNAi significantly reduced *daf-2(e1370)* freeze–thaw survival, although there was still higher survival than in the *daf-16*;*daf-2* double mutant (Fig. 8). This result indicates that the *daf-16* target genes *acs-19* and *srh-99* are involved in the observed *daf-2*(rf) increased freeze–thaw stress survival. While, there is no evidence indicating other daf-16 target genes *sup-37* or *lig-1* involved in *daf-2(rf)* increased freezing survival (Fig. 8).

## Discussion

In this study, we revealed genetic regulation of freeze–thaw stress responses in *C. elegans*. Phenotypic analysis of genetic deletion strains revealed that insulin/IGF-1 receptor *daf-2* controls both survival and behavior during freeze–thaw stress. *daf-2* reduction improved freeze–thaw stress survival, locomotion, and muscle cell protection. *daf-2*(rf) freeze–thaw response is highly allele-specific and not a consequence of lifespan, dauer formation, or other stress-resistance trait regulation. We also revealed that insulin signaling, but not TGF-β, are related to freeze–thaw survival. Steroid hormone signaling participated in *daf-2*(rf) enhanced freeze–thaw stress regulation. *daf-16*::GFP cellular localization analysis and *daf-16* target gene screening revealed that *daf-16* regulated the target genes *acs-19* and *srh-99* but not *daf-16* nuclear translocation or *daf-2*(rf) enhanced freeze–thaw stress survival.

In the past few decades, there has been rapid progress in our understanding of how physiological mechanisms can protect freeze injury [24]. By contrast, very little is known about how genetic regulation promotes freeze resistance. At least in *C. elegans*, freeze resistance is not purely a passive thermodynamic process. In this study, we characterized insulin/IGF-1 receptor *daf-2*, which regulates freeze–thaw stress survival improvement in *C. elegans*. Our results indicate that genetic programming actively contribute to enhanced survival and physiological function recovery from freezing conditions.

Previous work revealed that insulin signaling is required for lifespan regulation, dauer formation, and stress tolerance. Recent studies demonstrated that the insulin-signaling pathway or *daf-16* is required for temperature experience-dependent cold tolerance of animals [12, 13]. To ascertain whether insulin signaling plays roles in regulating freeze resistance in very stressful conditions, we performed survival experiments with freeze–thaw stress. In these experiments, we confirmed that insulin signaling via the *daf-16*/FOXO pathway is essential for freeze–thaw survival. Reduction-of-function of insulin/IGF-R *daf-2* protected muscle cell damage and promoted physiological activity recovery from freeze–thaw stress.

We found that freeze–thaw survival was *daf-2*(rf) allele-specific and is not a consequence of ageing, larval arrest, or other stress-resistance traits. Insulin signaling is also involved in dauer formation. To determine whether other dauer signaling participated in the freeze–thaw stress resistance, we assayed freeze–thaw survival of mutants. We found that dauer formation pathways, including TGF-β and steroid receptor signaling, were not essential for freeze–thaw stress survival. In addition, *daf-2* is the best-characterized longevity-regulated gene. Therefore, we also tested other freeze survival longevity gene mutants, including *eat-2* [25], *clk-1* [26], and *isp-1* [27]. We found that these other longevity genes were not required for freeze-stress resistance. Low temperatures led to increased longevity, in which the cold receptor TRP channel encoded by *trpa-1* is essential [21, 28]. Therefore, we tested the function of *trpa-1* and found that loss-of-function mutant *trpa-1* produced a normal phenotype compared with the wild-type strain. These results indicate that freeze–thaw survival is independent on cold receptor TRPA-1 signaling. These results are consistent with the *daf-2*(rf) allele relationship analysis between freeze–thaw survival and lifespan, larval arrest, and other stress–resistance traits. Freezing resistance may have an independent biological mechanism that differs from mechanisms that control ageing, dauer formation, and response to other stresses.

Finally, our results indicate that the *daf-16* target genes *C36A4.9* (*acs-19*) and *C46A10.7* (*srh-99*) are required for freezing survival of *daf-2(e1370)*. Previous studies demonstrated that *acs-19* maintained core body temperatures of mice in fasting conditions [22], and olfactory receptor be acclimated to the environmental temperature of *D. melanogaster* [23]. Inactivation by RNAi of *acs-19* or *srh-99* contributed to both decreased fat storage and enhanced dauer formation of *C. elegans* [29]. It is possible that an unrecognized underlying mechanism contributes to both fat storage and dauer formation phenomena, which are related to freezing tolerance and survival. Nevertheless, our analyses did not exclude the contribution of additional mechanisms to freezing survival. *acs-19* and *srh-99* RNAi reduced freezing survival of *daf-2(e1370)*; however, *daf-2(e1370) acs-19* RNAi and *daf-2(e1370) srh-99* RNAi mutants exhibited significantly higher survival compared with the *daf-16* mutant, which indicates that additional *daf-16* target genes or mechanisms must be involved. For example, genes involved in the synthesis of trehalose [30–34], glycerol [30], heat-shock proteins [32, 35, 36], antioxidant enzymes [37], and Δ9 desaturase enzymes [12] could participate in freeze–thaw stress survival by enhancing cold tolerance of *C. elegans* and other species. Future studies are needed to identify other unknown genetic programming and additional components of the *daf-2*(rf)/*daf-16* pathway that ultimately may lead to a thorough understanding of how *daf-2*(rf) promotes freezing survival in *C. elegans*.

## Conclusion

In conclusion, our mutant survival assay revealed that insulin/IGF-1 receptor *daf-2* played important roles in freeze–thaw stress responses in *C. elegans*. Freezing resistance is *daf-2* allele-specific and not a consequence of ageing, dauer formation, and other stress-regulation traits. Reduction-of-function of *daf-2* enhanced freeze–thaw survival, because it is dependent on insulin signaling pathway. The *daf-16*/FOXO-regulating target genes *acs-19* are *srh-99* essential for *daf-2*(rf) enhanced freezing resistance. Considering that the insulin/IGF-1 receptor showed striking conservation across phylogeny [38, 39], our work indicates that a similar phenomenon may also occur in other organisms.

## Methods

### Strain selection and maintenance

*Caenorhabditis elegans* were maintained on nematode growth medium (NGM) agar plates seeded with OP50, which is a slow-growing *Escherichia coli* mutant. The strains used included *N2*, *eat-2(ad465)*, *isp-1(qm150)*, *clk-1(qm30)*, *daf-1(m40)*, *daf-7(e1372)*, *daf-12(rh286)*, *daf-2(e1370)*, *daf-2(e979)*, *daf-2(e1391)*, *daf-2(e1368)*, *daf-2( e1371)*, *daf-2(m596)*, *daf-2(m577)*, *daf-2(m579)*, *daf-2(Sa193)*, *daf-2(m41)*, *[daf-16(mgDf47); daf-2(e1370)]*, *TJ356(daf-16::GFP)*, and *PD4251(pmyo-3::GFP).* All strains were provided by the Caenorhabditis Genetics Center funded by the National Institutes of Health National Center for Research Resources. Unless otherwise stated, all strains were cultured at 15 °C to the L4 stage and then transferred to animals at 20 °C for 2 d.

### Freeze–thaw stress conditions

Adult animals (2 d after L4) on NGM plates were washed with M9 buffer and transferred into FACS tube with 1 ml M9 buffer. *Caenorhabditis elegans* does not survive freezing very well, so to determine optimal freezing length for analyses, we placed animals from room temperature into −80 °C for different amounts of times (4 min, 5 min,6 min, 8 min, 12 min, or 16 min). At 4 min, there was no survival difference between *N2* and *daf-2(e1370)*. Ice began to form at 5 min, and all water froze in the tube at 6–7 min; however, all animals, including *N2* and *daf-2*(rf), died at 16 min. At 8 min, there were significant freezing survival differences among mutants. Therefore, animals were treated with freezing stress at −80 °C for 8 min for subsequent analyses. This experiment revealed that freezing stress damaged animals; it is possible that in the body, some cells did not completely freeze, but the freezing stress still injured animals.

Animals were exposed to freezing stress and then thawed at different temperatures (0 °C and 30 °C) for different lengths of time (10 min for 0 °C and approximately 1 min for 30 °C). We removed the tubes at the end of thawing process (when the ice was completely thawed), when the temperature in the tube is still 0 °C.

All treated animals were placed on dry NGM plates for 6 h before determining mortality score with an optical microscope. L1–adult animals were used for the *daf-2*(rf) survival test for different stages.

### Programmed freezing conditions

L1–adult animals on NGM plates were gently washed with M9 buffer. For the freezing procedure, a Cryo 1 °C freezing container was used with a gradient cooling rate of −1 °C/min. Animals were transferred into freezing tubes with 0.9 ml buffer and 0.9 ml 30 % glycerin. At different time points (every 20 or 40 min), animals were thawed in a 30 °C water bath, and the survival rate was then assayed.

### Feeding RNAi

dsRNA-expressing *E. coli* were streaked onto LB agar plates that contained ampicillin (50 μg ml^−1^) and tetracycline (12.5 μg ml^−1^), and then incubated at 37 °C overnight. Bacteria were inoculated in 3 ml LB liquid medium that contained only ampicillin (100 μg ml^−1^) and then incubated at 37 °C overnight. All 3 ml of culture was spun down, the supernatant poured off until 150 μl was left (20× concentrated culture), and pellets were resuspended. Then, 50 μl of cells were resuspended to the center of RNAi plate (NGM/IPTG/ampicillin), allowed to dry (wrapped in aluminum foil), and induced overnight at room temperature (RNAi-seeded plates can be stored at room temperature for 2–3 d before use). Synchronized L1 worms were placed on each plate and incubated at 15 or 20 °C until they reached the desired stage for further experiments.

### Scoring mortality

Worms were scored on NGM plates after 6 h of recovery from the thawing procedure. Worms were prodded with a pick at least three times over approximately 10 s; any that failed to move were counted as dead and removed from the plate.

### Morphologic cell defects

The *PD4251* reporter gene *pmyo-3*::*gfp*, which is located in body wall muscle nuclei, was assayed with a fluorescence microscope. *pmyo-3*::*gfp* expression of strain *PD4251* by RNAi treatment with freezing and thawing was assayed.

### Quantitation of locomotion rate by counting body bends

Ten animals were selected from fresh plates with fairly thin lawns, and one worm each was placed new plates. Assays started 24 h later (±1 h). A 3-min timer was used to count number of body bends. Every time the part of the worm just behind the pharynx reached a maximum bend in the opposite direction from the bend last counted was considered one body bend.

### *daf-16* nucleus translocation

*TJ356* nuclear protein *daf-16*::GFP expression was assayed with a fluorescence microscope. *daf-16*::GFP expression of [*daf-2*(RNAi); *TJ356*] treated with freezing and thawing was assayed compared with that of *TJ356*.

### RNA isolation for qRT-PCR

Total mRNA was extracted by TRIzol reagent (Invitrogen, Carlsbad, CA) from certain *C. elegans* and treated with RNase-free DNase (Promega, Madison, WI). Then, reverse transcription (RT) was performed with a TaKaRa RNA PCR kit (Takara, Dalian, China) following the manufacturer’s instructions. The primers used are described in Additional file 5: Table S1 and sequenced in the DNA Sequencing Department of Biosune Systems Biology (Shanghai, China).

### mRNA reverse transcription and qRT-PCR

One nanogram in vitro transcribed RNA was added to the RNA sample (500–1000 ng). DEPC H_2_O was added to the RNA sample to 29.5 μl. Then, 0.5 μl each of 1 μg/μl random hexamer and 1 μg/μl poly dT were added. The mixture was incubated at 65 °C for 10 min, immediately put on ice for 5 min, and let stand at room temperature for 10 min. Then, 18.5 μl pre-mixture, which contained 2.5 μl 10 mM dNTP mix, 10 μl 5× first-strand buffer, 5 μl 0.1 M DTT, and 1 μl RNase OUT, was added. The mixture was then mixed and spun, and placed at room temperature for 2 min. Then, 1 μl Superscript II RT was added and gently mixed. The mixture was then spun down and let stand at room temperature for 10 min, incubated at 42 °C for 50 min, and heat inactivated at 70 °C for 15 min. Then, 1 μl RNase H was added to the solution, which was gently mixed, spun down, and blocked at 37 °C for 30 min.

SYBR Green qRT-PCR was performed on the LightCycler® 480 II System (Roche, Pleasanton, CA) using 5 μl 2× SYBR Green master mix (Roche), 2 μl RNAse free water, 1 μl of forward, 1 μl of reverse primer (10 μM), and 1 μl cDNA per reaction. Primer efficiency was assessed by a dilution series, and a dissociation curve was used to assess primer specificity. qRT-PCR data analysis was performed using genEx software (MultiD).

### Statistical analyses

Percent survival was reported as mean ± SEM per trial. Every test was repeated at least three times under the same conditions. Survival rate was analyzed by the nonparametric Mann–Whitney test. Correlation analysis between *daf-2(rf)* allele freeze–thaw survival and lifespan/other stress traits were conducted by the nonparametric Spearman correlation test.

### Availability of supporting data

Primer sequences,data are accessible through this link: https://mynotebook.labarchives.com/share/fangny/MjMuNHwxMzY4MDYvMTgvVHJlZU5vZGUvMzYxNTQ3NzkyNXw1OS40.

## Additional files

Additional file 1: Figure S1.
*daf-2(e1370)* enhanced freezing survival under programmed freezing conditions. Reduction-of-function mutant *daf-2(e1370)* (squares) exhibited significantly increased survival compared with wild-type (*N2*) animals under the programmed cooling conditions (-1 °C/min) at different stages. (JPG 259 kb) Additional file 2: Figure S2. Survival rates of wild-type (*N2*) and *daf-2(e1370rf)* under different thawing processes. With different thawing treatments (30 °C water bath for 1 min or ice for 30 min), wild-type (*N2*) and *daf-2(e1370rf)* animals had unchanged survival rates after freeze–thaw stress. (JPG 94 kb) Additional file 3: Figure S3. Correlated analysis of *daf-2(rf)* freeze–thaw survival and lifespan, larval arrest, and other stress-resistance traits. Freeze–thaw stress survival phenotypes were not correlated with lifespan, larval arrest, or hypoxia resistance , but was moderately correlated with heat shock survival . (JPG 41 kb) Additional file 4: Figure S4. Screening for *daf-16* target genes required for freezing survival by QF-PCR. *daf-16* target genes C01B7.1(*sup-37*), C36A4.9 (*acs-19*) , C29A12.3(*lig-1*) and C46A10.7 (*srh-99*) have higher expression in *daf-2(e1370)* compared with wild-type (*N2*) animals. (JPG 92 kb) Additional file 5: Table S1.
*daf-2(rf)* allelic variation influence on freeze–thaw stress survival. (JPG 169 kb)
