# Supplementary figures and images for: Freeze–thaw Caenorhabditis elegans freeze–thaw stress response is regulated by the insulin/IGF-1 receptor daf-2
Source: BMC Genet. 2015 Dec 3;16:139. doi: 10.1186/s12863-015-0298-5 (PMC4669615; doi:10.1186/s12863-015-0298-5)

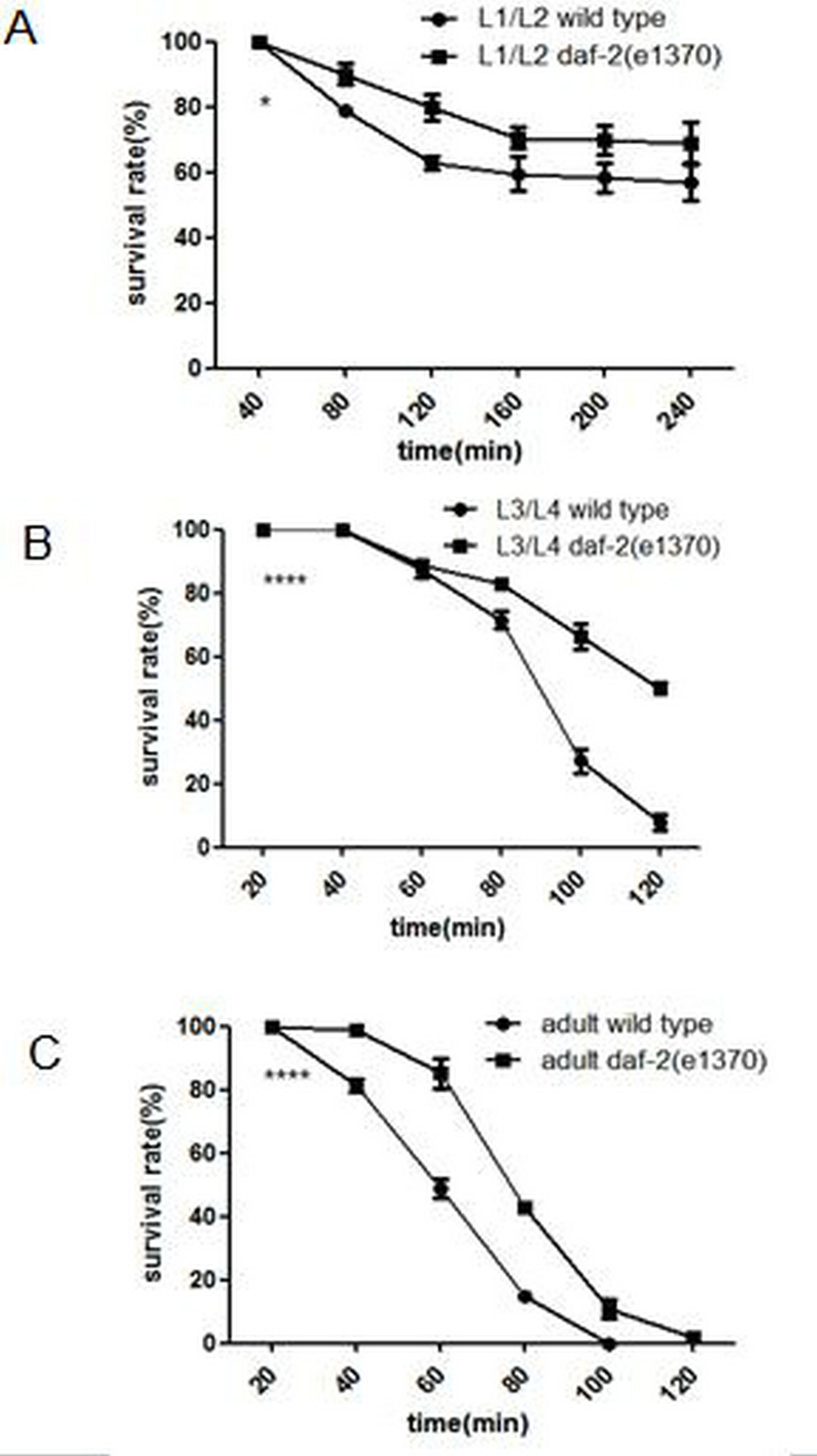

Supplement: Additional file 1: Figure S1. — daf-2(e1370) enhanced freezing survival under programmed freezing conditions. Reduction-of-function mutant daf-2(e1370) (squares) exhibited significantly increased survival compared with wild-type (N2) animals under the programmed cooling conditions (-1 °C/min) at different stages. (JPG 259 kb) [file 12863_2015_298_MOESM1_ESM.jpg]

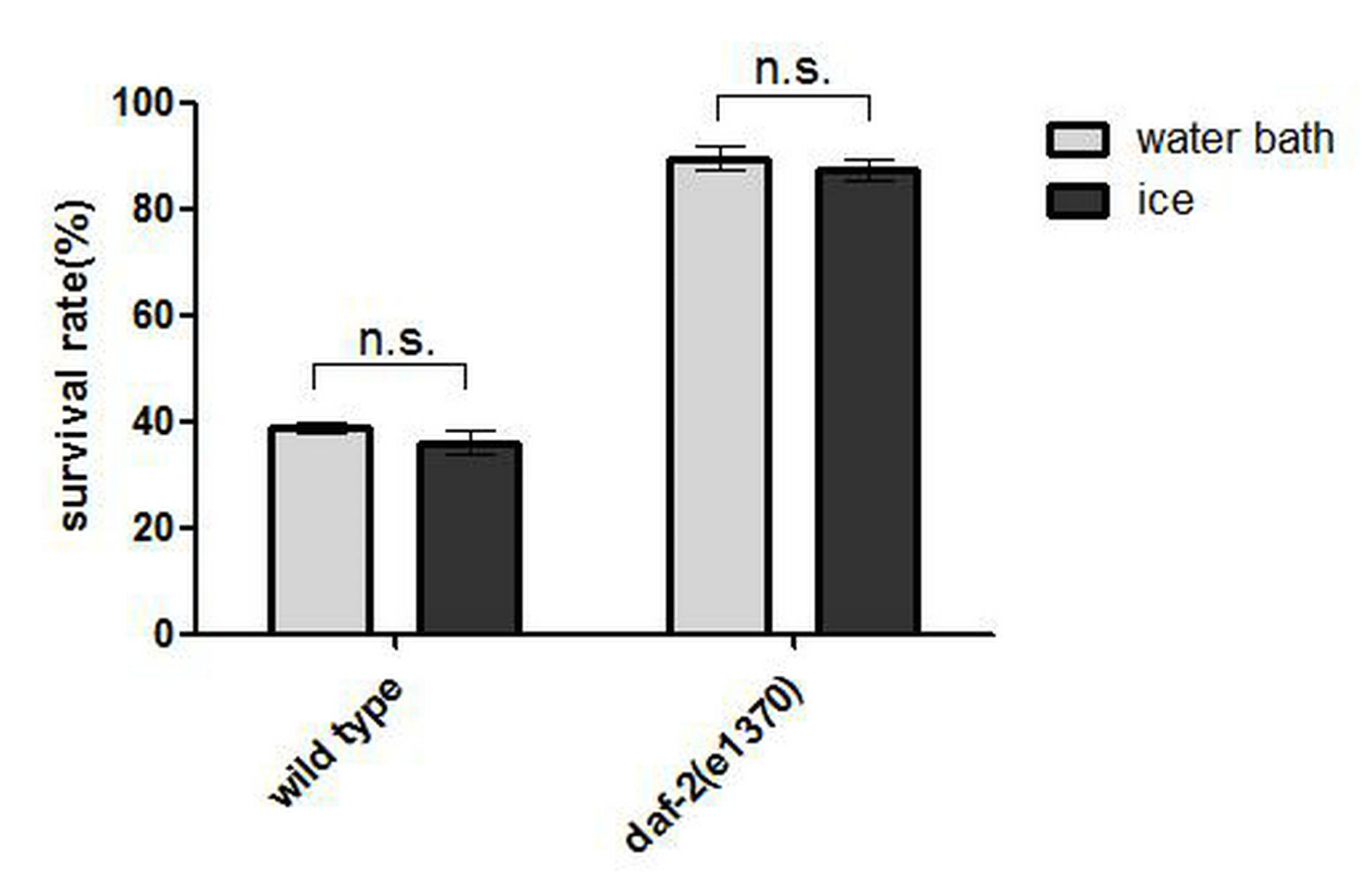

Supplement: Additional file 2: Figure S2. — Survival rates of wild-type (N2) and daf-2(e1370rf) under different thawing processes. With different thawing treatments (30 °C water bath for 1 min or ice for 30 min), wild-type (N2) and daf-2(e1370rf) animals had unchanged survival rates after freeze–thaw stress. (JPG 94 kb) [file 12863_2015_298_MOESM2_ESM.jpg]

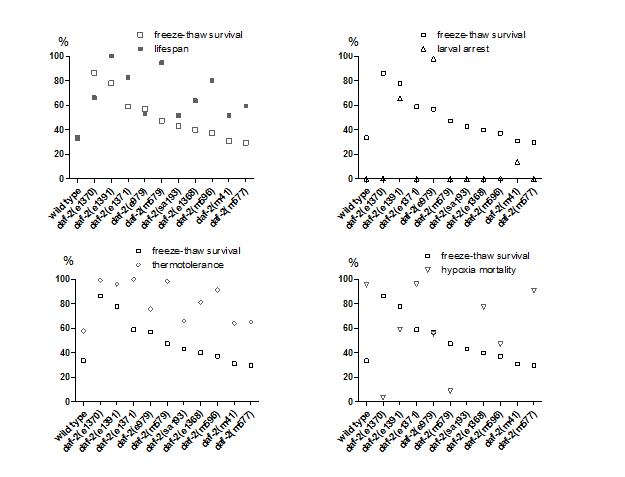

Supplement: Additional file 3: Figure S3. — Correlated analysis of daf-2(rf) freeze–thaw survival and lifespan, larval arrest, and other stress-resistance traits. Freeze–thaw stress survival phenotypes were not correlated with lifespan, larval arrest, or hypoxia resistance , but was moderately correlated with heat shock survival . (JPG 41 kb) [file 12863_2015_298_MOESM3_ESM.jpg]

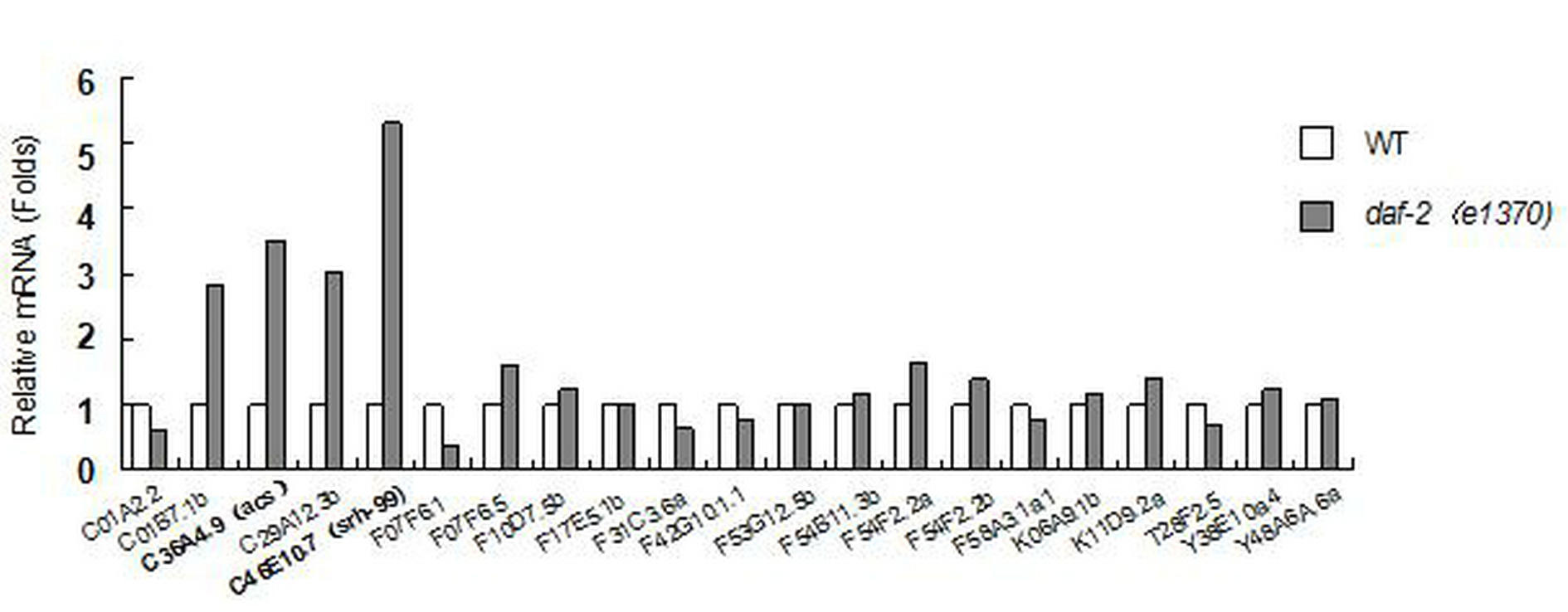

Supplement: Additional file 4: Figure S4. — Screening for daf-16 target genes required for freezing survival by QF-PCR. daf-16 target genes C01B7.1(sup-37), C36A4.9 (acs-19) , C29A12.3(lig-1) and C46A10.7 (srh-99) have higher expression in daf-2(e1370) compared with wild-type (N2) animals. (JPG 92 kb) [file 12863_2015_298_MOESM4_ESM.jpg]

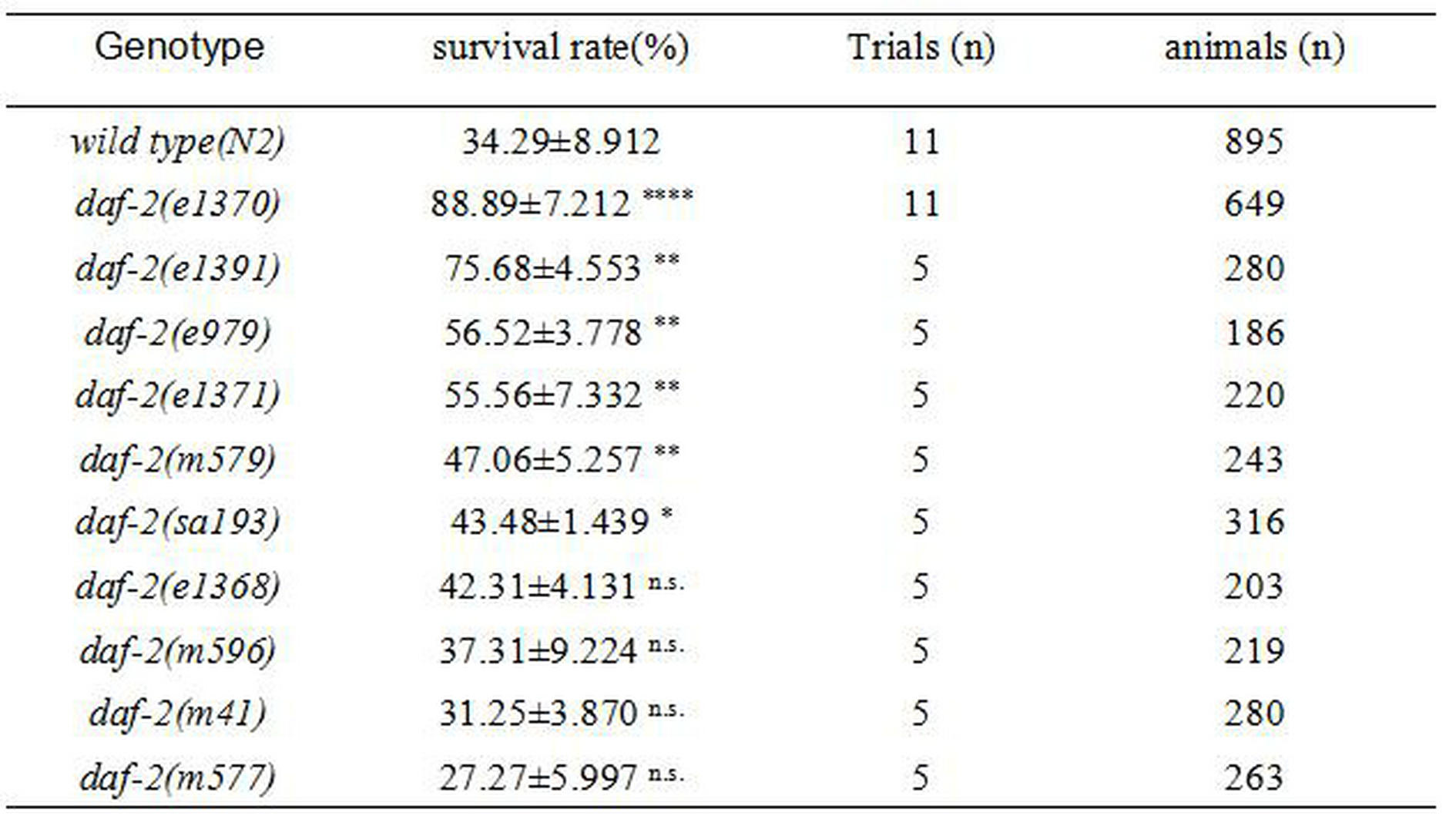

Supplement: Additional file 5: Table S1. — daf-2(rf) allelic variation influence on freeze–thaw stress survival. (JPG 169 kb) [file 12863_2015_298_MOESM5_ESM.jpg]
